# A stochastic SIR epidemic model with Lévy jump and media coverage

**DOI:** 10.1186/s13662-020-2521-6

**Published:** 2020-02-12

**Authors:** Yingfen Liu, Yan Zhang, Qingyun Wang

**Affiliations:** 1 College of Mathematics and Computer Science, Gannan Normal University, Ganzhou, P.R. China; 2 School of Mathematics and Statistics, Wuhan University, Wuhan, P.R. China

**Keywords:** Lévy jump, Temporary immunity, Threshold value, Extinction

## Abstract

A stochastic susceptible–infectious–recovered epidemic model with temporary immunity and media coverage is proposed. The effects of Lévy jumps on the dynamics of the model are considered. A unique global positive solution for the epidemic model is obtained. Sufficient conditions are derived to guarantee that the epidemic disease is extinct and persistent in the mean. The threshold behavior is discussed. Numerical simulations are given to verify our theoretical results.

## Introduction

Epidemics have a huge impact on human life, and controlling and eradicating infectious diseases have been a vital problem that needs to be urgently solved in eco-epidemiology research. Mathematical modeling has become an important tool in analyzing the spread and control of infectious diseases. In implementing measures for preventing the spread of diseases, educating people about the correct preventions of diseases through mass media and other platforms at the first opportunity is particularly important [1]. The coverage of epidemics in the media, such as through television, newspaper, and online networks, gives an overview of the risk level and the relative need for precautions in risk areas and encourages the public to take precautionary measures, such as wearing masks, avoiding public places, and frequent hand washing [2]. Thus, in the past few years, many epidemic models integrating the effects of media coverage have been presented and analyzed [3–11].

Temporary immunity is another important phenomenon in the transmission of epidemic diseases, such as influenza, Chlamydia trachomatis, and Salmonella infection [12]. In the case of temporary immunity, an individual gets a fleeting immunity to a disease after recovery and then becomes susceptible again after some period. For example, after recovery from influenza, there is a long immunity to the same strain of the disease but no immunity against other strains. Many scholars have also paid close attention to the effects of temporary disease immunity on epidemic models [13–18]; however, only a few have considered the effects of media coverage and temporary immunity simultaneously.

On the basis of the aforementioned discussion, a deterministic susceptible–infectious–recovered (SIR) model that considered media coverage and temporary immunity is proposed as follows:

1.1 $$ \textstyle\begin{cases} \frac{\mathrm{d}S}{\mathrm{d}t}= \varLambda -\mu S(t)- (\beta _{1}-\frac{\beta _{2}I}{\alpha +I} )S(t)I(t)+ \gamma I(t- \tau )e^{-\mu \tau }, \\ \frac{\mathrm{d}I}{\mathrm{d}t}= (\beta _{1}-\frac{\beta _{2}I}{\alpha +I} )S(t)I(t)-(\mu +\gamma )I(t), \\ \frac{\mathrm{d}R}{\mathrm{d}t}= \gamma I(t)-\gamma I(t-\tau )e^{-\mu \tau }-\mu R(t), \end{cases} $$

where *Λ* is the recruitment rate, *μ* denotes the natural death rate, and *γ* is the treatment rate. $\tau >0$ is the length of temporary immunity period, which denotes the time from recovery to becoming susceptible again. The term $I(t-\tau )e^{- \mu \tau }$ reflects the fact that an individual has survived from natural death in a recovery pool before becoming susceptible again [13]. $\beta =\beta _{1}-\frac{\beta _{2}I(t)}{ \alpha +I(t)}$ denotes the effective contact rate, here, $\beta _{1}$ represents the maximal effective contact rate between susceptible and infected individuals, $\frac{\beta _{2}I(t)}{\alpha +I(t)}$ is the maximal reduced effective contact rate as influenced by mass media alert [5, 6]. $\alpha >0$ is the effect of media coverage on contact transmission, and $\beta _{1}>\beta _{2}$.

On the other hand, epidemic models are inevitably subject to environmental noise and it is necessary to reveal how the environmental noise affects the epidemic model. In the natural world, there are various types of random noises, such as the famous white noise, Lévy jump noise which considers the motivation that the continuity of solutions may be broken under severe environmental perturbations, such as avian influenza, severe acute respiratory syndrome, volcanic eruptions, earthquakes, hurricanes [19–21] and a jump process should be introduced to prevent and control diseases, and so on. In this paper, we extend the deterministic system (1.1) to the Brown motion with Lévy jumps, $J(t)=\int _{0}^{t}\int _{\mathbf{Y}}\gamma (u) \tilde{N}(\mathrm{d}s,\mathrm{d}u)$, and mainly consider its effects on the effective contact rate parameter $\beta =\beta _{1}-\frac{\beta _{2}I}{\alpha +I}$ such that

$$ \beta \rightarrow \beta +\sigma \dot{B}(t)+\dot{J}(t). $$

Considering the effects of temporary immunity and media coverage on a stochastic susceptible–infectious–recovered (SIR) epidemic model driven by Lévy noise:

1.2 $$ \textstyle\begin{cases} {\mathrm{d}}S= [\varLambda -\mu S(t)- (\beta _{1}-\frac{\beta _{2}I}{\alpha +I} )S(t)I(t)+ \gamma I(t- \tau )e^{-\mu \tau } ]\, {\mathrm{d}}t \\ \hphantom{{\mathrm{d}}S= {}} {} - (\beta _{1}-\frac{\beta _{2}I}{\alpha +I} )S(t)I(t) ( \sigma \, {\mathrm{d}}B(t)+ \int _{\mathbf{Y}}\gamma (u)\tilde{N}(\mathrm{d}t,\mathrm{d}u) ) , \\ {\mathrm{d}}I= [ (\beta _{1}-\frac{\beta _{2}I}{\alpha +I} )S(t)I(t)-(\mu +\gamma )I(t) ]\, {\mathrm{d}}t+ (\beta _{1}-\frac{\beta _{2}I}{\alpha +I} )S(t)I(t) (\sigma \, { \mathrm{d}}B(t) \\ \hphantom{{\mathrm{d}}I={}} {}+ \int _{\mathbf{Y}}\gamma (u)\tilde{N}(\mathrm{d}t,\mathrm{d}u) ) , \\ {\mathrm{d}}R= [\gamma I(t)-\gamma I(t-\tau )e^{-\mu \tau }-\mu R(t) ]\, {\mathrm{d}}t. \end{cases} $$

The initial conditions are

1.3 $$ \begin{aligned} &S(0)=S_{0}\geq 0,\qquad I(\xi )=\phi _{1}(\xi )\geq 0, \\ &\phi _{1}(0)>0,\quad \xi \in [-\tau ,0], \phi _{1}\in C \bigl([-\tau ,0]; \mathbb{R} _{+} \bigr), \end{aligned} $$

where $\tau >0$ is the length of the temporary immunity period, which covers the time from recovery phase to being the susceptible ones again; and $\sigma ^{2}(t)$ denotes the intensity of white noise. $B(t)$ is a standard Brownian motion that is defined on a complete probability space $(\varOmega ,\mathcal{F},\mathbb{P})$ with filtration $\{\mathcal{F}_{t} \}_{t\in \mathbb{R}_{+}}$ satisfying the usual conditions ($\{ \mathcal{F}_{t}\}_{t\in \mathbb{R}_{+}}$ is right continuous and increasing while $\mathcal{F}_{0}$ contains all $\mathbb{P}$-null sets) [22–26]. *N* is a Poisson counting measure with compensator *Ñ* and characteristic measure *λ* on a measurable subset **Y** of $(0,\infty )$ which satisfies $\lambda (\mathbf{Y})<\infty $; *λ* is assumed to be a Lévy measure, such that $\tilde{N}(\mathrm{d}t,\mathrm{d}u)=N( \mathrm{d}t,\mathrm{d}u)-\lambda \tilde{N}(\mathrm{d}u)\,\mathrm{d}t$; $\gamma : \mathbf{Y}\times \varOmega \rightarrow \mathbb{R}$ is bounded and continuous with respect to *λ* and is $\mathfrak{B}(\mathbf{Y}) \times \mathcal{F}_{t}$-measurable, where $\mathfrak{B}(\mathbf{Y})$ is a *σ*-algebra with respect to the set **Y**. In this paper, *B* and *N* are assumed to be independent of each other.

As the first two equations of models (1.2) do not depend on the third one, then the following equations should be considered:

1.4 $$ \textstyle\begin{cases} {\mathrm{d}}S= [\varLambda -\mu S(t)- (\beta _{1}-\frac{\beta _{2}I}{\alpha +I} )S(t)I(t)+ \gamma I(t- \tau )e^{-\mu \tau } ]\, {\mathrm{d}}t \\ \hphantom{{\mathrm{d}}S={}} {} - (\beta _{1}-\frac{\beta _{2}I}{\alpha +I} )S(t)I(t) ( \sigma\, {\mathrm{d}}B(t)+ \int _{\mathbf{Y}}\gamma (u)\tilde{N}(\mathrm{d}t,\mathrm{d}u) ) , \\ {\mathrm{d}}I= [ (\beta _{1}-\frac{\beta _{2}I}{\alpha +I} )S(t)I(t)-(\mu +\gamma )I(t) ]\, {\mathrm{d}}t+ (\beta _{1}-\frac{\beta _{2}I}{\alpha +I} )S(t)I(t) (\sigma \, { \mathrm{d}}B(t) \\ \hphantom{{\mathrm{d}}I={}} {}+ \int _{\mathbf{Y}}\gamma (u)\tilde{N}(\mathrm{d}t,\mathrm{d}u) ). \end{cases} $$

Moreover, we make the following assumption.

### Assumption (H1)

$\gamma (u)$ is a bounded function, $1+\gamma (u)>0$ and $|\frac{\varLambda }{\mu }\gamma (u)|\leq \delta $, $u\in \mathbf{Y}$.

### Remark 1

This assumption means that the intensities of Lévy noises are not infinite.

The outline of this paper is as follows. In Sect. 2, a unique positive solution for system (1.4) is obtained. The conditions are derived for the extinction and persistence in the mean of diseases. The threshold behavior is obtained and discussed. In Sect. 3, some numerical simulations are presented to verify our theoretical results of system (1.4).

## Main results

### Existence and uniqueness of the global solution

In the following, we discuss the existence and uniqueness of the positive solution of system (1.4).

#### Theorem 2.1

*If Assumption *(H1)*holds*, *then*, *for any initial value*$(S(0), I(0))\in L^{1}([-\tau ,0]; \mathbb{R}^{2}_{+})$, *a unique solution*$(S(t), I(t))\in \mathbb{R}^{2}_{+}$*of system* (1.4) *exists on*$t\geq -\tau $*and the solution will remain in*$\mathbb{R} ^{2}_{+}$*with probability one*.

#### Proof

According to the local Lipschitz condition of system (1.4), we see that, for any initial value $X_{0}=(S(0), I(0))\in \mathbb{R}^{2}_{+}$, a unique local solution $(S(t), I(t))$ exists on $[-\tau , \tau _{e})$, herein, $\tau _{e}$ represents the explosion time. To prove that the solution is global, one is required to obtain $\tau _{e}=\infty $ a.s. Then we suppose that $k_{0}\geq 1$ is sufficiently large such that $S(0)$ and $I(0)$ lie within the interval $[1/k_{0},k_{0}]$. For each integer $k>k_{0}$, we define the stopping time $\tau _{k} =\inf \{t \in [-\tau ,\tau _{e}]: S(t) {\notin }(1/k,k), \text{or } I(t) {\notin }(1/k,k)\}$. Then $\tau _{k}$ increases as $k\rightarrow \infty $. Denote $\tau _{\infty }={\lim_{k\rightarrow +\infty }}\tau _{k}$, thus $\tau _{\infty }\leq \tau _{e}$. In the following, we need to show that $\tau _{\infty }=\infty $. If not, there are constants $T>0$ and $\varepsilon \in (0,1)$ satisfying ${P}\{\tau _{\infty }<\infty \}>\varepsilon $. Thus, an integer $k_{1}\geq k_{0}$ exists such that ${P}\{\tau _{k}\leq T\}\geq \varepsilon $, for all $k>k_{1}$. Construct a $C^{2}$-function $V: \mathbb{R}_{+}^{2}\rightarrow \mathbb{R}_{+}$ by

2.1 $$ V(S,I)= \biggl(S-a-a\ln \frac{S}{a} \biggr)+(I-1-\ln I)+\gamma e^{- \mu \tau } \int _{t-\tau }^{t}I(s)\,\mathrm{d}s, $$

where *a* is a constant that will be given later. By virtue of Itô’s formula, we have

2.2 $$\begin{aligned} {\mathrm{d}}V(S,I) =& \biggl(1-\frac{a}{S} \biggr) \biggl[ \biggl( \varLambda -\mu S- \biggl(\beta _{1}-\frac{\beta _{2}I}{ \alpha +I} \biggr)SI+ \gamma I(t-\tau )e^{-\mu \tau } \biggr)\,\mathrm{d}t \\ &{}-\sigma SI \biggl(\beta _{1}-\frac{ \beta _{2}I}{\alpha +I} \biggr)\, \mathrm{d}B_{1}(t) \biggr] +\frac{a\sigma ^{2}S^{2}I^{2}}{2S^{2}} \biggl(\beta _{1}-\frac{\beta _{2}I}{ \alpha +I} \biggr)^{2}{\mathrm{d}}t \\ &{}-a \int _{\mathbf{Y}} \biggl[\ln \biggl(1- \gamma (u) \biggl(\beta _{1}-\frac{\beta _{2}I}{\alpha +I} \biggr)I \biggr) +\gamma (u)I \biggl( \beta _{1}-\frac{\beta _{2}I}{\alpha +I} \biggr) \biggr]\lambda (\mathrm{d}u) \,\mathrm{d}t \\ &{} - \int _{\mathbf{Y}} \biggl[a\ln \biggl(1-\gamma (u) \biggl(\beta _{1}-\frac{\beta _{2}I}{\alpha +I} \biggr)I \biggr) +\gamma (u)SI \biggl( \beta _{1}-\frac{\beta _{2}I}{\alpha +I} \biggr) \biggr]\tilde{N}(\mathrm{d}t, \mathrm{d}u) \\ &{} + \biggl(1-\frac{1}{I} \biggr) \biggl[ \biggl( \biggl(\beta _{1}-\frac{\beta _{2}I}{\alpha +I} \biggr)SI-( \mu +\gamma )I \biggr)\, \mathrm{d}t+ \sigma \biggl(\beta _{1}-\frac{\beta _{2}I}{\alpha +I} \biggr)SI \,\mathrm{d}B(t) \biggr] \\ &{} - \int _{\mathbf{Y}} \biggl[\ln \biggl(1+\gamma (u) \biggl(\beta _{1}-\frac{\beta _{2}I}{\alpha +I} \biggr)S \biggr) -\gamma (u) \biggl(\beta _{1}- \frac{\beta _{2}I}{\alpha +I} \biggr)S \biggr]\lambda (\mathrm{d}u) \, \mathrm{d}t \\ &{}+\frac{ \sigma ^{2}S^{2}I^{2}}{2I^{2}} \biggl(\beta _{1}- \frac{\beta _{2}I}{\alpha +I} \biggr)^{2} {\mathrm{d}}t \\ &{} + \int _{\mathbf{Y}} \biggl[\gamma (u) \biggl(\beta _{1}- \frac{\beta _{2}I}{\alpha +I} \biggr)SI- \ln \biggl(1+\gamma (u) \biggl(\beta _{1}-\frac{\beta _{2}I}{\alpha +I} \biggr)S \biggr) \biggr] \tilde{N}( \mathrm{d}t,\mathrm{d}u) \\ &{} +\gamma Ie^{-\mu \tau }{\mathrm{d}}t-\gamma I(t-\tau )e^{-\mu \tau } {\mathrm{d}}t \\ =& \biggl(1-\frac{a}{S} \biggr) \biggl[ \biggl(\varLambda -\mu S- \biggl( \beta _{1}-\frac{\beta _{2}I}{ \alpha +I} \biggr)SI+\gamma I(t-\tau )e^{-\mu \tau } \biggr) \biggr]{\mathrm{d}}t \\ &{} + \biggl[\frac{a\sigma ^{2}S^{2}I^{2}}{2S^{2}} \biggl(\beta _{1}- \frac{\beta _{2}I}{ \alpha +I} \biggr)^{2}-a \int _{\mathbf{Y}} \biggl[\ln \biggl(1-\gamma (u) \biggl(\beta _{1}-\frac{\beta _{2}I}{\alpha +I} \biggr)I \biggr) \\ &{}+\gamma (u) \biggl(\beta _{1}-\frac{\beta _{2}I}{\alpha +I} \biggr)I \biggr]\lambda (\mathrm{d}u) \biggr]{\mathrm{d}}t \\ &{} + \biggl[ \biggl(1-\frac{1}{I} \biggr) \biggl( \biggl(\beta _{1}-\frac{\beta _{2}I}{\alpha +I} \biggr)SI-( \mu +\gamma )I \biggr)+ \frac{\sigma ^{2}S^{2}I^{2}}{2I^{2}} \biggl(\beta _{1}-\frac{ \beta _{2}I}{\alpha +I} \biggr)^{2} \biggr]{\mathrm{d}}t \\ &{} - \int _{\mathbf{Y}} \biggl[\ln \biggl(1+\gamma (u) \biggl(\beta _{1}-\frac{\beta _{2}I}{\alpha +I} \biggr)S \biggr) -\gamma (u) \biggl(\beta _{1}- \frac{\beta _{2}I}{\alpha +I} \biggr)S \biggr]\lambda (\mathrm{d}u) \, \mathrm{d}t \\ &{} +\gamma Ie^{-\mu \tau _{2}}-\gamma I(t-\tau _{2})e^{-\mu \tau _{2}}) \,\mathrm{d}t \\ &{} -\sigma \biggl(1-\frac{a}{S} \biggr) \biggl(\beta _{1}-\frac{\beta _{2}I}{\alpha +I} \biggr)SI \,\mathrm{d}B(t) +\sigma \biggl(1- \frac{1}{I} \biggr) \biggl(\beta _{1}- \frac{\beta _{2}I}{ \alpha +I} \biggr)SI\,\mathrm{d}B(t) \\ &{} - \int _{\mathbf{Y}} \biggl[a\ln \biggl(1-\gamma (u) \biggl(\beta _{1}-\frac{\beta _{2}I}{\alpha +I} \biggr)I \biggr) +\gamma (u)SI \biggl( \beta _{1}-\frac{\beta _{2}I}{\alpha +I} \biggr) \biggr]\tilde{N}(\mathrm{d}t, \mathrm{d}u) \\ &{} + \int _{\mathbf{Y}} \biggl[\gamma (u) \biggl(\beta _{1}- \frac{\beta _{2}I}{\alpha +I} \biggr)SI- \ln \biggl(1+\gamma (u) \biggl(\beta _{1}-\frac{\beta _{2}I}{\alpha +I} \biggr)S \biggr) \biggr] \tilde{N}( \mathrm{d}t,\mathrm{d}u) \\ =& LV(S,I)\,\mathrm{d}t -\sigma \biggl(1-\frac{a}{S} \biggr) \biggl( \beta _{1}-\frac{\beta _{2}I}{ \alpha +I} \biggr)SI\,\mathrm{d}B(t) \\ &{}+\sigma \biggl(1-\frac{1}{I} \biggr) \biggl(\beta _{1}- \frac{ \beta _{2}I}{\alpha +I} \biggr)SI\,\mathrm{d}B(t) \\ &{} - \int _{\mathbf{Y}} \biggl[a\ln \biggl(1-\gamma (u) \biggl(\beta _{1}-\frac{\beta _{2}I}{\alpha +I} \biggr)I \biggr) +\gamma (u)SI \biggl( \beta _{1}-\frac{\beta _{2}I}{\alpha +I} \biggr) \biggr]\tilde{N}(\mathrm{d}t, \mathrm{d}u) \\ &{} + \int _{\mathbf{Y}} \biggl[\gamma (u) \biggl(\beta _{1}- \frac{\beta _{2}I}{\alpha +I} \biggr)SI- \ln \biggl(1+\gamma (u) \biggl(\beta _{1}-\frac{\beta _{2}I}{\alpha +I} \biggr)S \biggr) \biggr] \tilde{N}( \mathrm{d}t,\mathrm{d}u). \end{aligned}$$

Here, $LV: \mathbb{R}^{2}_{+}\rightarrow \mathbb{R}_{+}$ is defined as follows:

2.3 $$\begin{aligned} LV(S,I) =& \biggl(1-\frac{a}{S} \biggr) \biggl[ \biggl( \varLambda -\mu S- \biggl(\beta _{1}-\frac{\beta _{2}I}{ \alpha +I} \biggr)SI+ \gamma I(t-\tau )e^{-\mu \tau } \biggr) \biggr] \\ &{} + \biggl[\frac{a\sigma ^{2}S^{2}I^{2}}{2S^{2}} \biggl(\beta _{1}- \frac{\beta _{2}I}{ \alpha +I} \biggr)^{2}-a \int _{\mathbf{Y}} \biggl[\ln \biggl(1-\gamma (u) \biggl(\beta _{1}-\frac{\beta _{2}I}{\alpha +I} \biggr)I \biggr) \\ &{}+\gamma (u) \biggl(\beta _{1}-\frac{\beta _{2}I}{\alpha +I} \biggr)I \biggr]\lambda (\mathrm{d}u) \biggr] \\ &{} + \biggl[ \biggl(1-\frac{1}{I} \biggr) \biggl( \biggl(\beta _{1}-\frac{\beta _{2}I}{\alpha +I} \biggr)SI-( \mu +\gamma )I \biggr)+ \frac{\sigma ^{2}S^{2}I^{2}}{2I^{2}} \biggl(\beta _{1}-\frac{ \beta _{2}I}{\alpha +I} \biggr)^{2} \biggr] \\ &{} - \int _{\mathbf{Y}} \biggl[\ln \biggl(1+\gamma (u) \biggl(\beta _{1}-\frac{\beta _{2}I}{\alpha +I} \biggr)S \biggr) -\gamma (u) \biggl(\beta _{1}- \frac{\beta _{2}I}{\alpha +I} \biggr)S \biggr]\lambda (\mathrm{d}u) \\ &{} +\gamma Ie^{-\mu \tau _{2}}-\gamma I(t-\tau _{2})e^{-\mu \tau _{2}} \\ \leq &(\varLambda +\mu a+\mu +\gamma )-\frac{a\varLambda }{S} + \biggl[a \frac{ \beta _{2}I}{\alpha +I}-\mu -\gamma \bigl(1-e^{-\mu \tau } \bigr) \biggr]I \\ &{} +\frac{a\sigma ^{2}S^{2}I^{2}}{2S^{2}} \biggl(\beta _{1}-\frac{\beta _{2}I}{ \alpha +I} \biggr)^{2} +\frac{\sigma ^{2}S^{2}I^{2}}{2I^{2}} \biggl(\beta _{1}- \frac{ \beta _{2}I}{\alpha +I} \biggr)^{2} \\ &{} -a \int _{\mathbf{Y}} \biggl[\ln \biggl(1-\gamma (u) \biggl(\beta _{1}-\frac{\beta _{2}I}{\alpha +I} \biggr)I \biggr) +\gamma (u) \biggl(\beta _{1}- \frac{\beta _{2}I}{\alpha +I} \biggr)I \biggr]\lambda (\mathrm{d}u) \\ &{} - \int _{\mathbf{Y}} \biggl[\ln \biggl(1+\gamma (u) \biggl(\beta _{1}-\frac{\beta _{2}I}{\alpha +I} \biggr)S \biggr) -\gamma (u) \biggl(\beta _{1}- \frac{\beta _{2}I}{\alpha +I} \biggr)S \biggr]\lambda (\mathrm{d}u) \\ \leq & (\varLambda +\mu a+\mu +\gamma )-\frac{a\varLambda }{S} + \biggl[a \frac{\beta _{2}I}{ \alpha +I}-\mu -\gamma \bigl(1-e^{-\mu \tau } \bigr) \biggr]I \\ &{}+ \frac{a\sigma ^{2}S^{2}I ^{2}}{2S^{2}} \biggl(\beta _{1}-\frac{\beta _{2}I}{\alpha +I} \biggr)^{2} \\ &{} +\frac{\sigma ^{2}S^{2}I^{2}}{2I^{2}} \biggl(\beta _{1}-\frac{\beta _{2}I}{ \alpha +I} \biggr)^{2} +a \int _{\mathbf{Y}}\varphi _{1}\lambda (\mathrm{d}u) + \int _{\mathbf{Y}}\varphi _{2}\lambda (\mathrm{d}u), \end{aligned}$$

where $\varphi _{1}=-\ln (1-\gamma (u)(\beta _{1}-\frac{\beta _{2}I}{\alpha +I})I) -\gamma (u)(\beta _{1}- \frac{\beta _{2}I}{\alpha +I})I$, $\varphi _{2}=-\ln (1+\gamma (u)(\beta _{1}-\frac{\beta _{2}I}{\alpha +I})S) +\gamma (u)(\beta _{1}-\frac{ \beta _{2}I}{\alpha +I})S$ and choose $a=\frac{\mu +\gamma (1-e^{- \mu \tau })}{\beta _{2}}$.

On the other hand, notice that $\mathrm{d}(S+I+ \gamma e^{-\mu t} \int _{t-\tau }^{t}e^{\mu S}I(s)\,\mathrm{d}s)=[\varLambda -\gamma I-\mu (S+I+ \gamma e^{-\mu t}\int _{t-\tau }^{t}e^{\mu S}I(s)\,\mathrm{d}s)]{\mathrm{d}}t$. Then

$$\begin{aligned}& S+I+ \gamma e^{-\mu t} \int _{t-\tau }^{t}e^{\mu S}I(s)\,\mathrm{d}s \\ & \quad \leq \frac{\varLambda }{\mu }+e^{-\mu t} \biggl[S(0)+I(0)+ \gamma \int _{-\tau } ^{0}e^{\mu S}I(s) \, \mathrm{d}s-\frac{\varLambda }{\mu } \biggr] \\ & \quad \leq \textstyle\begin{cases} \frac{\varLambda }{\mu },& \text{if } S(0)+I(0)+ \gamma \int _{-\tau }^{0}e^{\mu S}I(s)\,\mathrm{d}s \leq \frac{\varLambda }{\mu }, \\ S(0)+I(0)+ \gamma \int _{-\tau }^{0}e^{\mu S}I(s)\,\mathrm{d}s, &\text{if } S(0)+I(0)+ \gamma \int _{-\tau }^{0}e^{\mu S}I(s) \,\mathrm{d}s>\frac{\varLambda }{\mu } \end{cases}\displaystyle \\ & \quad \triangleq K. \end{aligned}$$

Then applying the Taylor formula to the function $\ln (1-t)$ where $t=(\beta _{1}-\frac{\beta _{2}I}{\alpha +I})I\gamma (u)$ and Assumption (H1) to $\varphi _{1}$, we have

$$\begin{aligned} \varphi _{1} =& -\ln \biggl(1-\gamma (u) \biggl(\beta _{1}-\frac{\beta _{2}I}{\alpha +I} \biggr)I \biggr) - \gamma (u) \biggl(\beta _{1}-\frac{\beta _{2}I}{\alpha +I} \biggr)I \\ =& \gamma (u) \biggl(\beta _{1}-\frac{\beta _{2}I}{\alpha +I} \biggr)I+ \frac{(( \beta _{1}-\frac{\beta _{2}I}{\alpha +I})I\gamma (u))^{2}}{2(1-\theta \gamma (u)(2\beta _{1}-\beta _{2})I )^{2}}-\gamma (u) \biggl(\beta _{1}- \frac{ \beta _{2}I}{\alpha +I} \biggr)I \\ \leq & \frac{(2\beta _{1}-\beta _{2})^{2}\delta ^{2} }{2(1-(2\beta _{1}-\beta _{2})\delta )^{2}}, \end{aligned}$$

where $\theta \in (0,1)$ is an arbitrary number. Similarly,

$$\begin{aligned} \varphi _{2} =&- \int _{\mathbf{Y}} \biggl[\ln \biggl(1+\gamma (u) \biggl(\beta _{1}-\frac{ \beta _{2}I}{\alpha +I} \biggr)S \biggr) -\gamma (u) \biggl(\beta _{1}-\frac{\beta _{2}I}{ \alpha +I} \biggr)S \biggr]\lambda (\mathrm{d}u) \\ \leq& \frac{(2\beta _{1}-\beta _{2})^{2} \delta ^{2} }{2(1-(2\beta _{1}-\beta _{2})\delta )^{2}}. \end{aligned}$$

Then

$$\begin{aligned} LV(S,I) \leq & (\varLambda +\mu a+\mu +\gamma )+\frac{a\sigma ^{2}K^{2}}{2}(2\beta _{1}- \beta _{2})^{2} +\frac{\sigma ^{2}K^{2}}{2}(2 \beta _{1}-\beta _{2})^{2} \\ &{} +\frac{(a+1)\delta ^{2}}{2}\frac{(2\beta _{1}-\beta _{2})^{2}}{2(1- \delta (2\beta _{1}-\beta _{2}))^{2}}\triangleq \widetilde{K}. \end{aligned}$$

Therefore, we obtain

2.4 $$\begin{aligned} {\mathrm{d}}V(S, I) \leq & \widetilde{K} {\mathrm{d}}t-\sigma \biggl(1- \frac{a}{S} \biggr) \biggl(\beta _{1}-\frac{ \beta _{2}I}{\alpha +I} \biggr)SI\,\mathrm{d}B(t) +\sigma \biggl(1-\frac{1}{I} \biggr) \biggl( \beta _{1}-\frac{\beta _{2}I}{\alpha +I} \biggr)SI\,\mathrm{d}B(t) \\ &{} - \int _{\mathbf{Y}} \biggl[a\ln \biggl(1-\gamma (u) \biggl(\beta _{1}-\frac{\beta _{2}I}{\alpha +I} \biggr)I \biggr) +\gamma (u)SI \biggl( \beta _{1}-\frac{\beta _{2}I}{\alpha +I} \biggr) \biggr]\tilde{N}(\mathrm{d}t, \mathrm{d}u) \\ &{} + \int _{\mathbf{Y}} \biggl[\gamma (u) \biggl(\beta _{1}- \frac{\beta _{2}I}{\alpha +I} \biggr)SI- \ln \biggl(1+\gamma (u) \biggl(\beta _{1}-\frac{\beta _{2}I}{\alpha +I} \biggr)S \biggr) \biggr] \tilde{N}( \mathrm{d}t,\mathrm{d}u). \end{aligned}$$

Taking the integral on the above inequality from 0 to $\tau _{k} \wedge T$,

2.5 $$\begin{aligned}& \int _{0}^{\tau _{k}\wedge T}{\mathrm{d}}V(S, I) \\& \quad \leq \int _{0}^{\tau _{k}\wedge T}\widetilde{K} {\mathrm{d}}t - \int _{0}^{\tau _{k}\wedge T}\sigma \biggl(1- \frac{a}{S} \biggr) \biggl(\beta _{1}-\frac{\beta _{2}I}{ \alpha +I} \biggr)SI\,\mathrm{d}B(t) \\& \qquad {} + \int _{0}^{\tau _{k}\wedge T}\sigma \biggl(1- \frac{1}{I} \biggr) \biggl(\beta _{1}-\frac{ \beta _{2}I}{\alpha +I} \biggr)SI\,\mathrm{d}B(t) \\& \qquad {} - \int _{0}^{\tau _{k}\wedge T} \int _{\mathbf{Y}} \biggl[a\ln \biggl(1-\gamma (u) \biggl( \beta _{1}-\frac{\beta _{2}I}{\alpha +I} \biggr)I \biggr) +\gamma (u)SI \biggl( \beta _{1}-\frac{ \beta _{2}I}{\alpha +I} \biggr) \biggr]\tilde{N}(\mathrm{d}s, \mathrm{d}u) \\& \qquad {} + \int _{0}^{\tau _{k}\wedge T} \int _{\mathbf{Y}} \biggl[\gamma (u) \biggl(\beta _{1}- \frac{ \beta _{2}I}{\alpha +I} \biggr)SI-\ln \biggl(1+\gamma (u) \biggl(\beta _{1}-\frac{ \beta _{2}I}{\alpha +I} \biggr)S \biggr) \biggr]\tilde{N}( \mathrm{d}s,\mathrm{d}u), \end{aligned}$$

where $\tau _{k}\wedge T =\min \{\tau _{k}, T\}$. Consequently,

$$ EV \bigl(S(\tau _{k}\wedge T), I(\tau _{k}\wedge T) \bigr)\leq V \bigl(S(0), I(0) \bigr)+ \widetilde{K}E(\tau _{k}\wedge T) \leq V \bigl(S(0), I(0) \bigr)+\widetilde{K}T. $$

Let $\varOmega _{k}=\{\tau _{k}\leq T\}$, then ${P}(\varOmega _{k})\geq \varepsilon $. For each $\omega \in \varOmega _{k}$, $S(\tau _{k},\omega )$, or $I(\tau _{k},\omega )$, equals either *k* or $1/k$, and

$$ V \bigl(S(\tau _{k},\omega ), I(\tau _{k},\omega ) \bigr)\geq \min \{k-1- \ln k, 1/k-1+\ln k\}. $$

Thus,

2.6 $$\begin{aligned} V \bigl(S(0), I(0) \bigr)+K T \geq & E \bigl[1_{\varOmega _{k}}(\omega )V \bigl(S( \omega ), I(\omega ) \bigr) \bigr] \\ \geq & \varepsilon \min \{k-1-\ln k, 1/k-1+\ln k\}, \end{aligned}$$

where $1_{\varOmega _{k}}$ is the indicator function of $\varOmega _{k}$. Letting $k \rightarrow \infty $, we obtain the contradiction.

The proof is completed. □

### The extinction of diseases of system (1.4) with Lévy jumps

In this section, we define

$$ R_{0}=\frac{\varLambda (2\beta _{1}-\beta _{2})}{\mu (\mu +\gamma )}, $$

and denote $\langle x(t)\rangle =\frac{1}{t}\int _{0}^{t}x(s) \,\mathrm{d}s$, then the extinction of the disease will be discussed in the following.

#### Theorem 2.2

*Suppose*$(S(t), I(t))$*be any solution of system* (1.4) *with an initial value* (1.3). *Thus*:

1. *if*$\hat{\sigma }^{2}>\frac{(2\beta _{1}-\beta _{2})^{2}}{4( \mu +\gamma )}$, *then*
  $$ \limsup_{t\rightarrow \infty }\frac{\ln I(t)}{t}\leq \frac{(2 \beta _{1}-\beta _{2})^{2}}{4\hat{\sigma }^{2}}-( \mu +\gamma )< 0 \quad \textit{a.s.}; $$
2. *if*$R_{0}-1<\frac{\varLambda ^{2}\hat{\sigma }^{2}}{ \mu ^{2}(\mu +\gamma )}$*and*$\hat{\sigma }^{2}\leq \frac{\mu (2\beta _{1}-\beta _{2})}{2\varLambda }$, *then*
  $$ \limsup_{t\rightarrow \infty }\frac{\ln I(t)}{t}\leq (\mu + \gamma ) \biggl(R_{0}-1-\frac{\varLambda ^{2}\hat{\sigma }^{2}}{\mu ^{2}(\mu + \gamma )} \biggr)< 0\quad \textit{a.s.}, $$

*where*$\hat{\sigma }^{2}=\frac{\beta _{1}^{2}\alpha ^{2}\sigma ^{2}}{2( \alpha +N_{0})^{2}}+\int _{\mathbf{Y}}\frac{\gamma ^{2}(u)\frac{\beta _{1}\alpha }{\alpha +N_{0}}}{2(1+(2\beta _{1}-\beta _{2})\delta )^{2}} \lambda (\mathrm{d}u)$.

#### Proof

Applying Itô’s formula, we derive that

2.7 $$\begin{aligned} {\mathrm{d \ln }}I(t) =& \biggl[ \biggl(\beta _{1}- \frac{\beta _{2}I}{\alpha +I} \biggr)S-(\mu +\gamma )-\frac{\sigma ^{2}S^{2}}{2} \biggl(\beta _{1}-\frac{\beta _{2}I}{\alpha +I} \biggr)^{2} \biggr]\, { \mathrm{d}}t \\ &{}+\sigma \biggl(\beta _{1}-\frac{\beta _{2}I}{\alpha +I} \biggr)S \,\mathrm{d}B(t) \\ &{} + \int _{\mathbf{Y}} \biggl[\ln \biggl(1+ \biggl(\beta _{1}- \frac{\beta _{2}I}{ \alpha +I} \biggr)S\gamma (u) \biggr)- \biggl(\beta _{1}- \frac{\beta _{2}I}{\alpha +I} \biggr)S \gamma (u) \biggr] \lambda ( \mathrm{d}u) \\ &{}+ \int _{\mathbf{Y}}\ln \biggl(1+ \biggl(\beta _{1}- \frac{\beta _{2}I}{ \alpha +I} \biggr)S\gamma (u) \biggr)\tilde{N}(\mathrm{d}t, \mathrm{d}u). \end{aligned}$$

Then

2.8 $$\begin{aligned} \frac{\ln I(t)}{t} =& \frac{\ln I(0)}{t}+ \biggl\langle \biggl(\beta _{1}-\frac{\beta _{2}I}{ \alpha +I} \biggr)S \biggr\rangle -(\mu +\gamma ) \\ &{}- \frac{\sigma ^{2}}{2} \biggl\langle \biggl(\beta _{1}- \frac{\beta _{2}I}{\alpha +I} \biggr)^{2}S^{2} \biggr\rangle + \frac{M_{1}(t)}{t} +\frac{M_{2}(t)}{t} \\ &{} +\frac{1}{t} \int _{0}^{t} \int _{\mathbf{Y}} \biggl[\ln \biggl(1+ \biggl(\beta _{1}- \frac{ \beta _{2}I}{\alpha +I} \biggr)S\gamma (u) \biggr)- \biggl(\beta _{1}- \frac{\beta _{2}I}{ \alpha +I} \biggr)S\gamma (u) \biggr] \lambda ( \mathrm{d}u)\,\mathrm{d}s \\ \leq & (2\beta _{1}-\beta _{2}) \langle S\rangle -(\mu +\gamma )-\frac{\beta _{1}^{2}\alpha ^{2}}{2(\alpha +N_{0})^{2}}\sigma ^{2} \bigl\langle S^{2} \bigr\rangle +\frac{M_{1}(t)}{t} +\frac{M_{2}(t)}{t}+ \frac{\ln I(0)}{t} \\ &{} + \int _{\mathbf{Y}} \frac{\gamma ^{2}(u)\frac{\beta _{1}\alpha }{\alpha +N_{0}}}{2(1+(2\beta _{1}-\beta _{2})\delta )^{2}}\lambda (\mathrm{d}u) \bigl\langle S^{2} \bigr\rangle . \end{aligned}$$

Here, $M_{1}(t)=\int _{0}^{t}\sigma (\beta _{1}-\frac{\beta _{2}I}{ \alpha +I})S\,\mathrm{d}B(s)$ and $M_{2}(t)=\int _{0}^{t}\int _{ \mathbf{Y}}\ln (1+\gamma (u)(\beta _{1}-\frac{\beta _{2}I}{ \alpha +I})S)\tilde{N}(\mathrm{d}s, \mathrm{d}u)$.

On the other hand, we have

2.9 $$ {\mathrm{d}} \biggl(S+I+\gamma e^{-\mu \tau } \int _{t-\tau }^{t}I(s) \,\mathrm{d}s \biggr)= \bigl[ \varLambda -\mu S- \bigl(\mu +\gamma \bigl(1-e^{-\mu \tau } \bigr) \bigr)I \bigr]\, { \mathrm{d}}t. $$

Then

2.10 $$\begin{aligned}& \frac{S+I+\gamma e^{-\mu \tau }\int _{t-\tau }^{t}I(s)\,\mathrm{d}s}{t}-\frac{S(0)+I(0)+ \gamma e^{-\mu \tau }\int _{-\tau }^{0}I(s)\,\mathrm{d}s}{t} \\& \quad =\varLambda -\mu \bigl\langle S(t) \bigr\rangle - \bigl(\mu +\gamma \bigl(1-e^{-\mu \tau } \bigr) \bigr) \bigl\langle I(t) \bigr\rangle . \end{aligned}$$

Therefore,

2.11 $$ \bigl\langle S(t) \bigr\rangle =\frac{\varLambda }{\mu } -\frac{\mu +\gamma (1-e ^{-\mu \tau })}{\mu } \bigl\langle I(t) \bigr\rangle -\phi (t), $$

and here, $\phi (t)=\frac{S+I+\gamma e^{-\mu \tau }\int _{t-\tau }^{t}I(s) \,\mathrm{d}s}{\mu t} -\frac{S(0)+I(0)+\gamma e^{-\mu \tau }\int _{- \tau }^{0}I(s)\,\mathrm{d}s}{\mu t}$, thus $\lim_{t\rightarrow \infty }\phi (t)=0$. According to (2.11), we obtain

2.12 $$\begin{aligned} \frac{\ln I(t)}{t} \leq & (2\beta _{1}-\beta _{2}) \bigl\langle S \bigl(t^{-} \bigr) \bigr\rangle -(\mu +\gamma ) - \hat{ \sigma }^{2} \bigl\langle S^{2} \bigl(t^{-} \bigr) \bigr\rangle +\frac{M_{1}(t)}{t}+ \frac{M _{2}(t)}{t} + \frac{\ln I(0)}{t} \\ \leq & (2\beta _{1}-\beta _{2}) \biggl[ \frac{\varLambda }{\mu }-\frac{\mu +\gamma (1-e ^{-\mu \tau })}{\mu } \bigl\langle I(t) \bigr\rangle -\phi (t) \biggr]-(\mu +\gamma ) \\ &{} - \hat{\sigma }^{2} \biggl[ \frac{\varLambda }{\mu }- \frac{\mu +\gamma (1-e^{- \mu \tau })}{\mu } \bigl\langle I(t) \bigr\rangle -\phi (t) \biggr]^{2}+\frac{\ln I(0)}{t}+ \frac{M_{1}(t)+M_{2}(t)}{t} \\ =& (\mu +\gamma ) \biggl(\frac{\varLambda (2\beta _{1}-\beta _{2})}{\mu (\mu + \gamma )}-1- \frac{\varLambda ^{2}\hat{\sigma }^{2}}{\mu ^{2}(\mu +\gamma )} \biggr) \\ &{}-\frac{ \mu +\gamma (1-e^{-\mu \tau })}{\mu } \biggl((2\beta _{1}-\beta _{2})-2 \hat{\sigma }^{2}\frac{\varLambda }{\mu } \biggr) \bigl\langle I(t) \bigr\rangle \\ &{}+\frac{M_{1}(t)}{t}+\frac{M_{2}(t)}{t}+\psi (t), \end{aligned}$$

where

$$ \psi (t)= -(2\beta _{1}-\beta _{2})\phi (t)+ 2\hat{ \sigma }^{2}\frac{\varLambda }{ \mu }\phi (t)-\hat{\sigma }^{2} \biggl(\frac{\mu +\gamma (1-e^{-\mu \tau })}{\mu } \bigl\langle I(t) \bigr\rangle + \phi (t) \biggr)^{2}+\frac{\ln I(0)}{t}. $$

In addition,

$$\begin{aligned}& \langle M_{1},M_{1}\rangle _{t}=\sigma ^{2} \int _{0}^{t} \biggl(\beta _{1}- \frac{ \beta _{2}I}{\alpha +I} \biggr)^{2}S^{2}\, {\mathrm{d}}s, \\& \langle M_{2},M_{2}\rangle _{t}= \int _{0}^{t} \int _{\mathbf{Y}} \biggl( \ln \biggl(1+ \biggl(\beta _{1}-\frac{\beta _{2}I}{\alpha +I} \biggr)S\gamma (u) \biggr) \biggr)^{2}\lambda (\mathrm{d}u)\,\mathrm{d}s, \end{aligned}$$

and

$$ \ln \biggl(1+\frac{\beta _{1}\alpha }{\alpha +N_{0}}\theta \biggr)\leq \ln \biggl(1+ \biggl(\beta _{1}-\frac{\beta _{2}I}{\alpha +I} \biggr)S\gamma (u) \biggr) \leq \ln \bigl(1+(2\beta _{1}-\beta _{2})\theta \bigr). $$

Then we have

$$ \langle M_{2},M_{2}\rangle _{t}\leq \max \biggl\{ \bigl(\ln \bigl(1+(2\beta _{1}- \beta _{2})\theta \bigr) \bigr)^{2}, \biggl(\ln \biggl(1+\frac{\beta _{1}\alpha }{ \alpha +N_{0}}\theta \biggr) \biggr)^{2} \biggr\} \lambda (\mathbf{Y})t $$

and

$$\begin{aligned}& \limsup_{t\rightarrow \infty } \frac{\langle M_{1},M_{1}\rangle _{t}}{t}=\sigma ^{2} \limsup_{t\rightarrow \infty }\frac{1}{t} \int _{0}^{t} \biggl(\beta _{1}- \frac{ \beta _{2}I}{\alpha +I} \biggr)^{2}S^{2}{\mathrm{d}}s\leq \sigma ^{2}(2\beta _{1}-\beta _{2})^{2} \biggl(\frac{\varLambda }{\mu } \biggr)^{2} \\& \hphantom{\limsup_{t\rightarrow \infty } \frac{\langle M_{1},M_{1}\rangle _{t}}{t}}< \infty \quad \mbox{a.s.}, \\& \limsup_{t\rightarrow \infty } \frac{\langle M_{2},M_{2}\rangle _{t}}{t}\leq \max \biggl\{ \bigl( \ln \bigl(1+(2 \beta _{1}-\beta _{2})\theta \bigr) \bigr)^{2}, \biggl(\ln \biggl(1+\frac{\beta _{1} \alpha }{\alpha +N_{0}}\theta \biggr) \biggr)^{2} \biggr\} \lambda (\mathbf{Y}) \\& \hphantom{\limsup_{t\rightarrow \infty } \frac{\langle M_{2},M_{2}\rangle _{t}}{t}}< \infty , \quad \mbox{a.s.} \end{aligned}$$

Thus,

2.13 $$ \limsup_{t\rightarrow \infty }\frac{M_{i}(t)}{t}=0\quad (i=1,2) \quad \text{and}\quad \limsup_{t\rightarrow \infty }\psi (t)=0. $$

By virtue of the condition (2) and (2.12), we obtain

$$ \limsup_{t\rightarrow \infty }\frac{\ln I(t)}{t}\leq (\mu + \gamma ) \biggl(R_{0}-1-\frac{\varLambda ^{2}\hat{\sigma }^{2}}{\mu ^{2}(\mu + \gamma )} \biggr)< 0\quad \mbox{a.s.} $$

Moreover, according to (2.12), we have

2.14 $$\begin{aligned} \frac{\ln I(t)}{t} \leq & (2\beta _{1}-\beta _{2}) \bigl\langle S \bigl(t^{-} \bigr) \bigr\rangle -(\mu +\gamma )- \hat{ \sigma }^{2} \bigl\langle S \bigl(t^{-} \bigr) \bigr\rangle ^{2}+\frac{M_{1}(t)}{t}+\frac{M _{2}(t)}{t}+ \frac{\ln I(0)}{t} \\ =& -\hat{\sigma }^{2} \biggl[ \bigl\langle S \bigl(t^{-} \bigr) \bigr\rangle ^{2}-\frac{(2\beta _{1}- \beta _{2})}{\hat{\sigma }^{2}} \bigl\langle S \bigl(t^{-} \bigr) \bigr\rangle \biggr]-(\mu + \gamma ) + \frac{M_{1}(t)}{t}+\frac{M_{2}(t)}{t}+ \frac{\ln I(0)}{t} \\ =& -\hat{\sigma }^{2} \biggl( \bigl\langle S \bigl(t^{-} \bigr) \bigr\rangle -\frac{(2\beta _{1}-\beta _{2})}{2\hat{\sigma }^{2}} \biggr)^{2} + \frac{(2\beta _{1}-\beta _{2})^{2}}{4 \hat{\sigma }^{2}}-(\mu +\gamma ) \\ &{} +\frac{M_{1}(t)}{t}+\frac{M_{2}(t)}{t}+\frac{\ln I(0)}{t} \\ \leq & -(\mu +\gamma )+\frac{(2\beta _{1}-\beta _{2})^{2}}{4\hat{\sigma }^{2}} +\frac{M_{1}(t)}{t}+ \frac{M_{2}(t)}{t}+\frac{\ln I(0)}{t}. \end{aligned}$$

According to the condition (1) and (2.14), we obtain

$$ \limsup_{t\rightarrow \infty }\frac{\ln I(t)}{t}\leq -(\mu + \gamma )+ \frac{(2\beta _{1}-\beta _{2})^{2}}{4\hat{\sigma }^{2}}< 0, \quad \mbox{a.s.} $$

That is, $\lim_{t\rightarrow \infty }I(t)=0$. Moreover, we have

$$ \lim _{t\rightarrow \infty } \bigl\langle S(t) \bigr\rangle =\frac{\varLambda }{\mu } - \frac{\mu +\gamma (1-e^{-\mu \tau })}{\mu }\lim _{t\rightarrow \infty } \bigl\langle I(t) \bigr\rangle -\lim _{t\rightarrow \infty } \phi (t)=\frac{\varLambda }{\mu }. $$

The conclusion is proven. □

### Persistence in the mean of system (1.4)

Now we are in a position to discuss the persistence in the mean of the disease and before that some notations are presented in the following.

For convenience, we denote

$$\begin{aligned}& R_{1}=\frac{\beta _{1}\alpha \varLambda }{(\mu +\gamma )\mu (\alpha +N _{0})}, \\& \tilde{\sigma }=\frac{\sigma ^{2}}{2} \biggl(\frac{\beta _{1}\alpha }{\alpha +N _{0}} \biggr)^{2}N^{2}_{0} + \int _{\mathbf{Y}}\frac{(2\beta _{1}-\beta _{2})^{2} \delta ^{2}}{2(1-\delta (2\beta _{1}-\beta _{2}))^{2}}\lambda ( \mathrm{d}u), \\& \lambda ^{*}=(\mu +\gamma ) \biggl(R_{0}-1- \frac{\hat{\sigma }^{2}\varLambda ^{2}}{\mu ^{2}(\mu +\gamma )} \biggr), \\& \lambda _{0}=\frac{\mu +\gamma (1-e^{-\mu \tau })}{\mu } \biggl((2\beta _{1}- \beta _{2})-2\hat{\sigma }^{2}\frac{\varLambda }{\mu } \biggr), \\& I_{*}=\frac{\lambda ^{*}}{\lambda _{0}},\qquad \widetilde{I^{*}}= \frac{ \mu (\alpha +N_{0})((\mu +\gamma )(R_{1}-1)-\tilde{\sigma })}{\beta _{1}\alpha (\mu +\gamma (1-e^{-\mu \tau }))}. \end{aligned}$$

#### Theorem 2.3

*Suppose that Assumption *(H1)*holds and*$R_{1}-1>\frac{\tilde{\sigma }^{2}}{\mu +\gamma }$, *then*, *for the solution*$(S(t),I(t))$*of model* (1.4), *we have*

$$ \limsup_{t\rightarrow \infty } \bigl\langle I(t) \bigr\rangle \leq I_{*},\qquad \liminf_{t\rightarrow \infty } \bigl\langle I(t) \bigr\rangle \geq \widetilde{I^{*}}. $$

#### Proof

By virtue of (2.12), we have

2.15 $$\begin{aligned} \frac{\ln I(t)}{t} \leq & (\mu +\gamma ) \biggl(R_{0}-1- \frac{\varLambda ^{2}\hat{\sigma }^{2}}{\mu ^{2}(\mu +\gamma )} \biggr) -\frac{\mu +\gamma (1-e^{-\mu \tau })}{ \mu } \biggl((2\beta _{1}-\beta _{2})-2\hat{\sigma }^{2} \frac{\varLambda }{\mu } \biggr) \bigl\langle I(t) \bigr\rangle \\ &{} + \frac{M_{1}(t)}{t}+\frac{M_{2}(t)}{t}+\psi (t). \end{aligned}$$

Then

2.16 $$ \ln I(t)\leq \lambda ^{*} t-\lambda _{0} \int _{0}^{t}I(s)\,\mathrm{d}s+F(t), $$

here, $F(t)=M_{1}(t)+M_{2}(t)+\psi (t) t$.

Considering $\lim_{t\rightarrow \infty }\frac{F(t)}{t}=0$, then, for an arbitrary $\zeta >0$, there exist a $T_{1}=T_{1}(\omega )>0$ and a set $\varOmega _{k}$ such that $\frac{F(t)}{t}\leq \zeta $ and $P(\varOmega _{k})\geq 1-\zeta $ for all $t\geq T_{1}$, $\omega \in \varOmega _{k}$. Let $\hat{T}=\max \{T, T_{1}\}$, then according to Lemma 2.2 and Theorem 3 in Ref. [19], we obtain

2.17 $$ \limsup_{t\rightarrow \infty } \bigl\langle I(t) \bigr\rangle \leq \frac{\lambda ^{*}}{\lambda _{0}}\triangleq I_{*}. $$

On the other hand, by (2.8) and (2.11), we obtain

2.18 $$\begin{aligned} \frac{\ln I(t)}{t} =& \biggl\langle \biggl(\beta _{1}- \frac{\beta _{2}I}{\alpha +I} \biggr) S \biggr\rangle -(\mu + \gamma )- \frac{\sigma ^{2}}{2} \biggl\langle \biggl(\beta _{1}- \frac{\beta _{2}I}{ \alpha +I} \biggr)^{2} S^{2} \biggr\rangle \\ &{}+ \frac{M_{1}(t)}{t} + \frac{M_{2}(t)}{t}+\frac{ \ln I(0)}{t} \\ &{} +\frac{1}{t} \int _{0}^{t} \int _{\mathbf{Y}} \biggl[\ln (1+ \biggl(\beta _{1}- \frac{ \beta _{2}I}{\alpha +I} \biggr) S\gamma (u) - \biggl(\beta _{1}- \frac{\beta _{2}I}{ \alpha +I} \biggr) S\gamma (u) \biggr]\lambda (\mathrm{d}u)\, \mathrm{d}s \\ \geq & \frac{\beta _{1}\alpha }{\alpha +N_{0}} \bigl\langle S(t) \bigr\rangle -(\mu + \gamma )- \tilde{\sigma } +\frac{M_{1}(t)}{t} +\frac{M_{2}(t)}{t}+ \frac{ \ln I(0)}{t} \\ =& \frac{\beta _{1}\alpha }{\alpha +N_{0}} \biggl[\frac{\varLambda }{\mu }-\frac{ \mu +\gamma (1-e^{-\mu \tau })}{\mu } \bigl\langle I(t) \bigr\rangle -\phi (t) \biggr] \\ &{} -(\mu +\gamma )-\tilde{\sigma } + \frac{M_{1}(t)+M_{2}(t)+\ln I(0)}{t} \\ =& (\mu +\gamma ) \biggl[ \frac{\beta _{1}\alpha \varLambda }{\mu (\alpha +N _{0})(\mu +\gamma )} -1 \biggr] -\tilde{\sigma }- \frac{\beta _{1}\alpha ( \mu +\gamma (1-e^{-\mu \tau }))}{\mu (\alpha +N_{0})} \bigl\langle I(t) \bigr\rangle \\ &{} -\frac{\beta _{1}\alpha }{\alpha +N_{0}}\phi (t) +\frac{M_{1}(t)+M_{2}(t)+ \ln I(0)}{t} \\ =& (\mu +\gamma ) [ R_{1} -1 ] -\tilde{\sigma }- \frac{\beta _{1} \alpha (\mu +\gamma (1-e^{-\mu \tau }))}{\mu (\alpha +N_{0})} \bigl\langle I(t) \bigr\rangle -\frac{\beta _{1}\alpha }{\alpha +N_{0}}\phi (t) \\ &{} +\frac{M_{1}(t)+M_{2}(t)+\ln I(0)}{t}. \end{aligned}$$

As $0< S+I\leq N_{0}$, then we derive that $-\infty <\ln I(t)< \ln (N_{0})$. Thus,

2.19 $$\begin{aligned} \bigl\langle I(t) \bigr\rangle \geq & \frac{\mu (\alpha +N_{0})}{\beta _{1}\alpha (\mu +\gamma (1-e^{-\mu \tau }))} \biggl((\mu +\gamma ) (R_{1}-1)-\tilde{\sigma } \\ &{} -\frac{\beta _{1}\alpha }{\alpha +N_{0}}\phi (t)+ \frac{M_{1}(t)+M_{2}(t)}{t} - \frac{\ln (N_{0})-\ln I(0)}{t} \biggr). \end{aligned}$$

By virtue of the conclusion $\lim_{t\rightarrow \infty }\phi (t)=0$, we derive that

2.20 $$ \liminf_{t\rightarrow \infty } \bigl\langle I(t) \bigr\rangle \geq \frac{\mu ( \alpha +N_{0}) [(\mu +\gamma )(R_{1}-1)-\tilde{\sigma }]}{\beta _{1} \alpha (\mu +\gamma (1-e^{-\mu \tau }))}\triangleq \widetilde{I^{*}}. $$

This completes the proof. □

## Discussions and numerical simulations for system (1.4)

In this paper, we propose a stochastic SIR epidemic model that incorporate the effects of temporary immunity and media coverage. Some theoretical results are obtained with the influence of Lévy jumps. We prove that the system has a unique global solution at first. Then the conditions for extinction and persistence of the disease is derived. The results reveal that the intensity of Lévy noises can greatly influence the extinction and persistence of the disease.

In the following, we give some numerical simulations to support our obtained theoretical results of model (1.4) through the Milstein method [27] and Euler numerical approximation [28].

### Example 3.1

**Figure 1:**
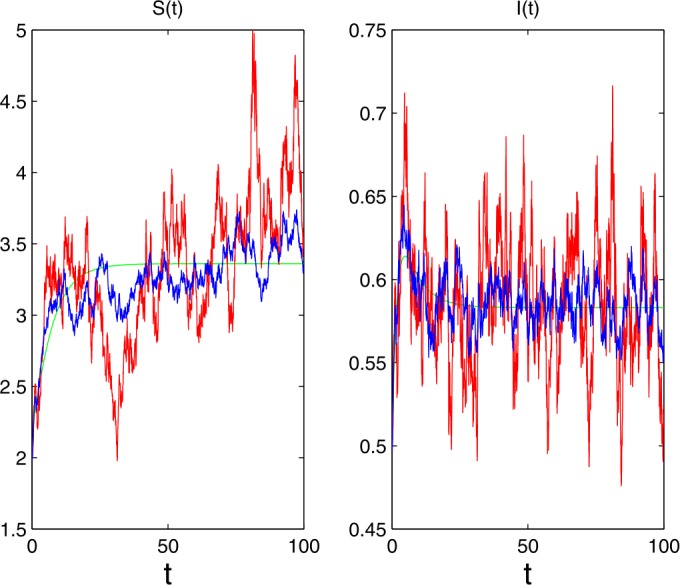
The populations are persistent in the mean for system (1.4)

Choose the parameter values in model (1.2) as follows:

$$\begin{aligned}& \varLambda =0.6,\qquad \beta _{1}=0.3,\qquad \beta _{2}=0.2, \qquad \mu =0.15, \qquad \gamma =0.2, \\& \alpha =1.8,\qquad \gamma (u)=0.07,\qquad \sigma =0.03,\qquad S(0)=2, \\& I(0)=0.5,\qquad \mathbf{Y}=(0,+\infty ),\qquad \lambda (\mathbf{Y})=1, \end{aligned}$$

then we have

$$ R_{1}=1.064>1+\frac{\tilde{\sigma }^{2}}{\mu +\gamma }=1.0251, $$

and the condition of Theorem 2.3 is satisfied. Thus, the disease *I* is persistent with probability one and Fig. 1 confirms it. The red lines, the green lines and the blue lines are solutions of system (1.4), the corresponding deterministic system and the system with white noise, respectively.

### Example 3.2

**Figure 2:**
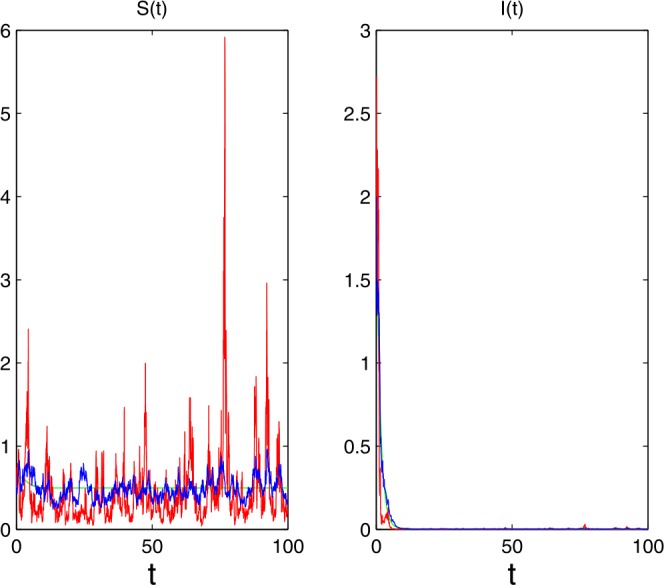
The disease goes to extinction

Let the parameters be as follows:

$$\begin{aligned}& \varLambda =0.3,\qquad \beta _{1}=0.002,\qquad \beta _{2}=0.001, \qquad \mu =0.6, \qquad \gamma =0.1,\qquad \alpha =0.1, \\& \sigma =0.3,\qquad \gamma (u)=0.6,\qquad S(0)=0.5,\qquad I(0)=2,\qquad \mathbf{Y}=(0,+\infty ),\qquad \lambda (\mathbf{Y})=1, \end{aligned}$$

then $R_{0}=0.0021< 1+\frac{\varLambda ^{2}\hat{\sigma }^{2}}{\mu ^{2}( \mu +\gamma )}\doteq 1$ and $\hat{\sigma }^{2}=1.3023*10^{-5}\leq \frac{ \mu (2\beta _{1}-\beta _{2})}{2\varLambda }=2.7*10^{-4}$. Applying the conditions (2) in Theorem 2.2, we derive that the infective population $I(t)$ will be extinct with probability one (see Fig. 2).

### Example 3.3

In model (1.4), set

$$ \varLambda =0.3,\qquad \beta _{1}=0.002,\qquad \beta _{2}=0.001, \qquad \mu =0.15, \qquad \gamma =0.01,\qquad \alpha =0.01. $$

**Figure 3:**
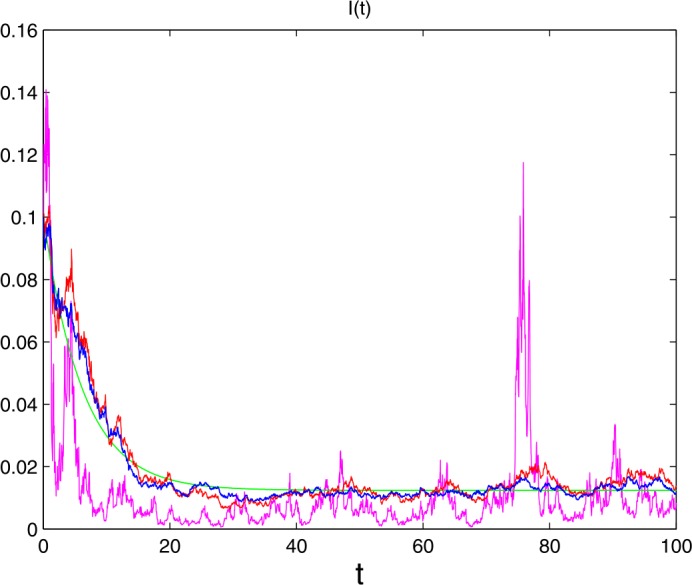
The effects of jumps to system (1.4)

The initial value is $(S(0), I(0))=(0.5, 0.1)$. To show the effects of noise to the system (1.4), two cases are considered as follows: (1) $\sigma =0.1$, $\gamma (u)=0.1$, (2) $\sigma =0.1$, $\gamma (u)=0.7$, and we obtain Fig. 3, where the green lines, the blue lines the red lines, and the rose lines denote solutions of the deterministic system, the system with white noise, system (1.4) with $\gamma (u)=0.1$ and $\gamma (u)=0.7$, respectively. We derive that jumps have negative effects for the prevailing of diseases (see Fig. 3).

At last, some interesting issues merit further investigations. In this paper, the threshold behavior is discussed and two threshold expressions $R_{0}$ and $R_{1}$ are obtained. However, the threshold value cannot be derived according to the complex expression of the contact rate and it is an interesting issue left for further work. Moreover, in this paper, we consider the effects of white noise and the Lévy jumps to the model behavior, however, if we also take other perturbations, such as the regime-switching [29–31] to the proposal of epidemic model, what will happen? We will also investigate this question in our future work.
